# Periodontitis and risk of stroke: a systematic review and meta-analysis of observational studies

**DOI:** 10.3389/fneur.2025.1700946

**Published:** 2025-11-03

**Authors:** Xinyu Meng, Xiaohua Chen

**Affiliations:** ^1^College of Stomatology, Binzhou Medical University, Yantai, Shandong, China; ^2^Department of Nuclear Medicine, Weifang People's Hospital, Weifang, Shandong, China

**Keywords:** periodontitis, stroke risk assessment, systematic review, meta-analysis, ischemic stroke

## Abstract

**Objectives:**

To evaluate whether periodontitis is associated with stroke and to update pooled estimates with recent studies.

**Methods:**

We systematically searched Medline, Embase, and the Cochrane Database of Systematic Reviews from inception to July 2025. We included cohort and case–control studies of adults comparing periodontitis with no periodontitis and reporting any stroke (ischemic, hemorrhagic, or unspecified). Random-effects models were used. Case–control and cohort data were pooled separately. Prespecified subgroups included stroke subtype, study design, sex, and follow-up duration. Publication bias was assessed when ≥10 studies were available.

**Results:**

Twenty-two studies were included (16 cohorts; 6 case–control). In case–control studies, periodontitis was associated with higher odds of stroke (OR, 2.22; 95% CI, 1.48–3.34; *I*^2^ = 33%). In cohorts, periodontitis was associated with increased incident stroke risk (RR, 1.49; 95% CI, 1.23–1.80; *I*^2^ = 96%). In subgroup analysis, prospective cohorts showed slightly higher and more stable estimates than retrospective cohorts (RR, 1.53 vs. 1.40; *I*^2^ = 61% vs. 98%). Studies with >10 years of follow-up showed a stronger, less heterogeneous association (RR, 1.57; 95% CI, 1.35–1.84; *I*^2^ = 32%). Funnel plots suggested limited publication bias.

**Conclusions:**

Periodontitis is associated with increased stroke risk, most clearly for ischemic outcomes and in long-term prospective cohorts. Given high heterogeneity and potential residual confounding, the findings support association rather than causation. Standardized periodontal definitions, subtype-specific endpoints, and rigorous prospective and interventional studies are needed to test clinical impact.

## Introduction

Stroke is the leading cause of long-term disability and the second leading cause of mortality across the world (1). The incidence continues to rise, with millions of new cases reported annually in both developed and developing countries (2). Traditional risk factors such as hypertension, diabetes, smoking, and obesity are well established, but they do not fully explain the occurrence of stroke. Increasing attention has been directed toward chronic inflammatory conditions as potential contributors to stroke (3, 4).

Periodontitis, one of the most prevalent chronic inflammatory diseases, affects more than 10% of people globally (5). It is initiated by a dysbiotic oral microbiota, notably pathogens such as Porphyromonas gingivalis and Fusobacterium nucleatum, which release virulence factors that trigger systemic inflammation and endothelial dysfunction (6). Elevated levels of C-reactive protein, interleukin-6, and tumor necrosis factor-α in patients with periodontitis provide a biological link to atherosclerosis and thrombus formation (6, 7). Recent studies also implicate the oral–gut–brain axis, whereby oral microbial dysbiosis modulates gut homeostasis and immune responses, further amplifying neuroinflammation in stroke (6, 8, 9).

Epidemiology supports an association between periodontitis and stroke, with pooled relative risks ranging from 1.22 to 2.88 in previous meta-analyses (10, 11). However, findings are not uniform and heterogeneity is substantial, as limited numbers of studies and variability in study design, diagnostic criteria, and confounder adjustment have led to inconsistent results. These controversies highlight the need for an updated synthesis, especially as new studies have been published. Therefore, we conducted a systematic review and meta-analysis to quantify the association between periodontitis and stroke and to explore heterogeneity through subgroup analyses, with the aim of informing prevention and clinical practice.

## Materials and methods

### Data source and search strategy

This systematic review and meta-analysis was conducted according to the Preferred Reporting Items for Systematic Reviews and Meta-Analyses (PRISMA) guidelines and was not previously registered. We systematically searched Medline (via Pubmed), Embase, and the Cochrane Database of Systematic Reviews (CDSR) from inception to July 2025. The search strategy was developed using the PICO framework: adults (Population), periodontitis (Exposure/Intervention), individuals without periodontitis (Comparison), and any stroke type (Outcome). Search terms included controlled vocabulary (e.g., MeSH, Emtree) and free-text words such as “periodontitis,” “periodontal disease,” “stroke,” “cerebrovascular accident,” “ischemic stroke,” and “hemorrhagic stroke.” The detailed strategies for each database are provided in the Supplementary Appendix. Additionally, reference lists of included studies and prior systematic reviews were screened manually to identify additional eligible publications. No restrictions on language or publication date were imposed.

### Study eligibility and selection

We included observational studies that evaluated periodontitis as a risk factor for stroke and reported extractable association measures. Eligible designs were cohort (prospective or retrospective, with ≥1 year follow-up) and case–control studies. Participants had to be adults (≥18 years) with periodontitis defined by clinical diagnosis (e.g., probing depth, clinical attachment loss, radiographic bone loss), validated indices, ICD codes, or confirmed self-report. Controls were individuals without periodontitis or with periodontal health. Outcomes of interest were incident or prevalent stroke events (ischemic, hemorrhagic, or unspecified), confirmed by medical records, imaging, ICD codes, or validated questionnaires.

We excluded studies that: (1) involved predominantly (>50%) children or adolescent populations; (2) evaluated gingivitis, tooth loss without attribution to periodontitis, or mixed periodontal conditions without stratification; (3) exposure assessment was not performed prior to the outcome or within a very short time window (for case-control studies) thereafter (e.g., within one week); (4) assessed cardiovascular events without stroke-specific outcomes; (5) used cross-sectional, ecological, case series, or case reports; (6) were reviews, guidelines, editorials, conference abstracts, or non–peer-reviewed sources; or (7) lacked sufficient data to compute association measures. When duplicate publications existed, we retained the study with the largest sample or most comprehensive analysis.

Two authors independently screened titles, abstracts, and full texts. Disagreements were resolved through consensus or by consulting an external expert.

### Data extraction and quality assessment

Two authors independently extracted data using a standardized form. Extracted information included: first author, publication year, country, study design, sample size, participant demographics, definition of periodontitis, number of teeth or sites examined, stroke subtype and diagnostic method, follow-up length, matching or adjustment variables, effect estimates (OR, RR, HR, and 95% CI), and funding source. When studies reported multiple models, we extracted the most fully adjusted estimates; if unavailable, unadjusted values were recorded.

Study quality was assessed using the Newcastle–Ottawa Scale (NOS) separately for cohort and case–control designs (12). The scale evaluates three domains: selection of study groups, comparability of groups, and ascertainment of exposure/outcome. Studies scoring >7 were considered high quality (low risk of bias), 5–7 moderate quality, and <5 low quality (high risk of bias). Disagreements were resolved by discussion or adjudication by an external expert.

### Data synthesis and analysis

We synthesized odds ratios (ORs) from case–control studies and relative risks (RRs), hazard ratios (HRs), or incidence rate ratios (IRRs) from cohort studies. In cohort studies, these effect estimates were treated as equivalent measures of relative risk on the logarithmic scale and combined using the generic inverse-variance (I-V) method. If cohort studies reported ORs, we converted them to RRs using the control group risk whenever available; when baseline risk was not reported and stroke events were rare, ORs were considered approximations of RRs, in line with Cochrane recommendations. Meta-analyses of case–control studies and cohort studies were conducted separately.

Random-effects models (DerSimonian–Laird method) were used to account for between-study variability. Statistical heterogeneity was quantified using the Q statistic and *I*^2^, with *I*^2^ >75% considered substantial (13). Publication bias was assessed with funnel plot symmetry when ≥10 studies were available, supplemented by Egger's regression test (14). We further applied the Trim and Fill method to adjust for any potential publication bias. This method imputes missing studies and recalculates the pooled effect size, providing a more accurate estimate in the presence of small-study effects or publication bias. All statistical tests were two-sided with a significance level of 0.05. Meta-analyses were performed using Review Manager (RevMan, version 5.4; Cochrane Collaboration).

Subgroup analyses were performed by stroke subtype (ischemic, hemorrhagic, unspecified), study design (prospective vs. Retrospective), sex, and mean follow-up duration. Sensitivity analyses were conducted by restricting to studies with multivariable-adjusted estimates and studies with low risk of bias. An additional sensitivity analyses was conducted by restricting to studies that diagnosed periodontitis through clinical examination or radiographic assessment.

## Results

**Figure 1:**
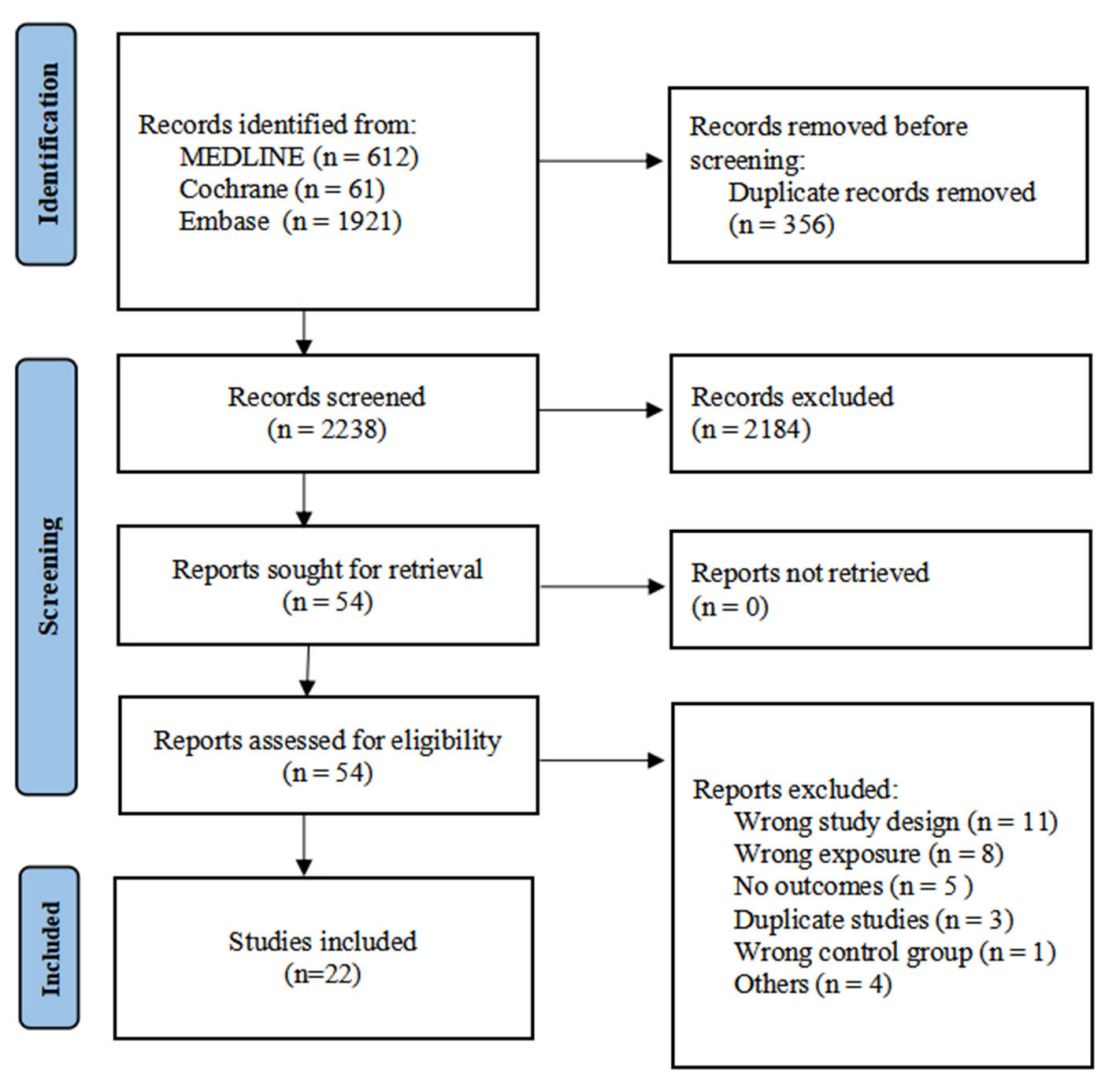
PRISMA flow diagram of study selection.

The database search identified 2,594 records. After removal of 356 duplicates, 2,238 records were screened, of which 2,184 were excluded based on title and abstract. Fifty-four full-text articles were assessed for eligibility. Twenty-two studies met the inclusion criteria and were included in the qualitative and quantitative synthesis (Figure 1).

### Study characteristics

A total of 22 observational studies were included, comprising 16 cohort studies (15–30) and 6 case–control studies (31–36), with study populations ranging from 200 to over 1.5 million participants. Geographically, the studies were conducted across multiple regions: North America, (15, 20, 22, 25, 28, 29) Europe, (16, 18, 19, 26, 30, 33, 34) Asia, (17, 23, 24, 27, 31, 35, 36) and Africa (32).

In total, the included cohorts contributed more than 2.3 million participants with follow-up durations ranging from 6 to 24 years. Periodontitis was most frequently assessed using radiographic bone loss or clinical attachment loss, while some studies employed alternative criteria, including ICD codes, questionnaires, and general practitioner reports, which may introduce variability in the definition of periodontitis. The number of teeth or sites examined varied substantially across studies. Stroke outcomes included ischemic, hemorrhagic, and unspecified stroke types, confirmed primarily through medical records, ICD codes, or neuroimaging. Most case–control studies matched cases and controls on age and sex, whereas some cohort studies reported additional adjustments for cardiovascular risk factors, socioeconomic status, or comorbidities. Detailed characteristics of the included studies are presented in Table 1.

**Table 1:** Characteristics of included studies.

**Author (Year), Country**	**Study design**	**Number of participants**	**Sex**	**Mean (median) age (years)**	**Matching**	**Periodontitis definition**	**Teeth/sites measured**	**Stroke type**	**Stroke ascertainment**	**Mean (median) follow-up length (years)**
Abolfazli 2011, Iran	Case-control	200	Mixed	54.2	Age, gender	Clinical attachment loss	All teeth, 4 sites/tooth	Ischemic stroke	CT/MRI	NA
Beck 1996, USA	Cohort (prospective)	1,147	Male	21–80	No	Radiographic bone loss	All teeth	Ischemic stroke	Clinical history/exam	18
Bengtsson 2021, Sweden	Cohort (prospective)	858	Mixed	72.0	No	Radiographic bone loss	Remaining teeth	Unspecified stroke	Medical records (ICD)	17
Diouf 2015, Senegal	Case-control	240	Mixed	NR	Age and sex	Clinical attachment loss	NR	NR	CT/MRI	NA
Dörfer 2004, Germany	Case-control	603	Mixed	58.4	Age and sex	Radiographic bone loss	All teeth, 4 sites/tooth	Ischemic stroke	CT/MRI	NA
Grau 2004, Germany	Case-control	771	Mixed	59.7	Age and sex	Clinical attachment loss	All teeth, 4 sites/tooth	Ischemic stroke	CT/MRI	NA
Hallikainen 2023, Finland	Cohort (prospective)	5,144	Mixed	>30	No	Other/unspecified	All teeth	Hemorrhagic stroke	Medical records (ICD)	13
Hansen, 2016, Denmark	Cohort (retrospective)	100,694	Mixed	57.3	Age, sex, and index date	Radiographic bone loss	NR	Ischemic stroke	Medical records (ICD)	15
Hashemipour 2013, Iran	Case-control	200	Mixed	51.9	Age and sex	Clinical attachment loss	All teeth	Ischemic stroke	CT/MRI	NA
Hsu 2022, China	Cohort (retrospective)	565,120	Mixed	0-80	Age, sex, urbanization level, income, and index day	Other/unspecified	NR	Not specified	Medical records (ICD)	≥5
Jimenez 2009, USA	Cohort (prospective)	1,137	Male	48	No	Radiographic bone loss	All teeth	Ischemic stroke	Medical records (ICD)	24
Joshipura (2003), United States	Cohort (prospective)	41,380	Male	40–75	No	Radiographic bone loss	NR	Ischemic stroke	Medical records (ICD)	12
LaMonte 2017, us	Cohort (prospective)	57,001	Female	68.1	No	Other/unspecified	NR	Ischemic stroke	Medical records (ICD)	6.7
Lee 2022, China Taiwan	Cohort(retrospective)	1,584,852	Mixed	37.26	Age- and sex	Other/unspecified	NR	Ischemic stroke	Medical records (ICD)	7.1 (mean), 14.28(median)
Lin 2019, Taiwan	Cohort(retrospective)	161,923	Mixed	46.7	No	Other/unspecified	NR	Not specified	Medical records (ICD)	7.74
Morrison 1999, Canada	Cohort(retrospective)	10,120	Mixed	35-84	No	Clinical attachment loss	NR	Not specified	Medical records (ICD)	23
Norhammar 2025, Sweden	Cohort (prospective)	1,587	Mixed	62	Age, gender, postal code area	Radiographic bone loss	All teeth	Not specified	Medical records (ICD)	9.9
Pradeep 2010, India	Case-control	200	Mixed	52.3	Age and gender	Clinical attachment loss	All teeth	Ischemic stroke	Medical records (ICD)	NA
Sen 2018, US	Cohort (prospective)	6,736	Mixed	62.3	No	Other/unspecified	All teeth	Ischemic stroke	Clinical diagnosis	15
Tiensripojamarn (2021), Thailand	cohort (prospective)	1,850	Mixed	58.6	No	Clinical attachment loss	All teeth	Not specified	Medical records (ICD)	13
Wu 2000, US	Cohort (prospective)	9,962	Mixed	45.26	No	Clinical attachment loss	NR	Ischemic + hemorrhagic	Medical records (ICD)	17–21
Zemedikun 2021, UK	Cohort(retrospective)	15,868	Mixed	44	Age, sex, Townsend Deprivation Index, registration date	Other/unspecified	NR	Not specified	Medical records (ICD)	6.23

### Quality assessment

Risk of bias was assessed using the Newcastle–Ottawa Scale (NOS). All 7 case–control studies were judged to be of high quality, with the main limitations related to non-response rate or ascertainment of exposure. For the 15 cohort studies, all achieved high quality ratings, with the main limitations related to non-response rate and selection of controls. A detailed summary of NOS assessments for each study is provided in Supplementary Tables 1, 2.

### Association between periodontitis and stroke

**Figure 2:**
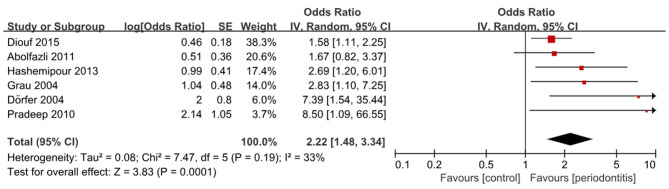
Forest plot for case-control studies on periodontitis and stroke association.

A total of 22 studies were eligible for quantitative synthesis, including 16 cohort and 6 case–control studies. Among the case–control studies, five demonstrated a significant association between periodontitis and stroke, (32–36) whereas one reported an OR greater than 1 that did not reach statistical significance (31). Pooled analysis confirmed that periodontitis was significantly associated with higher odds of stroke (6 studies; OR, 2.22; 95% CI, 1.48–3.34; *P* < 0.001; *I*^2^ = 33%) (Figure 2). Subgroup analysis by stroke sub-type demonstrated a stronger association for ischemic stroke (5 studies; OR, 2.66; 95% CI, 1.65–4.28; P <0.001; *I*^2^ = 12%) (Supplementary Table 3 and Supplementary Figure 1). More subgroup analysis are presented in Supplementary Figures 2, 3.

**Figure 3:**
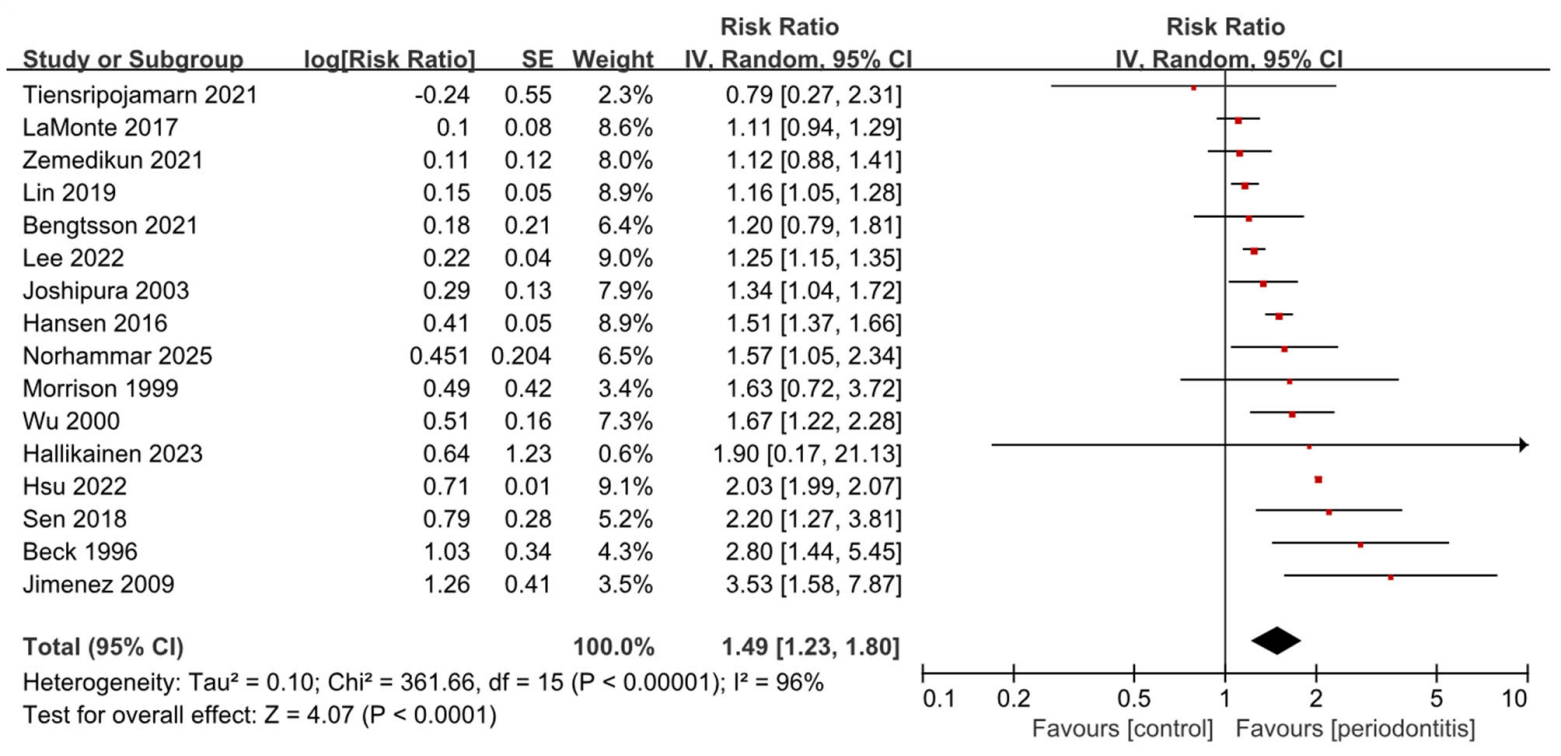
Forest plot for cohort studies on periodontitis and incident stroke.

In the cohort studies, ten reported a significant association between periodontitis and the risk of incident stroke, (15, 17, 19–21, 23, 24, 26, 28, 29) while five suggested an elevated risk that did not reach significance (16, 18, 22, 25, 30). Notably, a single study conducted in Thailand involving 1,850 participants found a lower stroke risk among individuals with periodontitis compared with controls, although the difference was not statistically significant (27). Overall, meta-analysis of cohort studies indicated that periodontitis was consistently associated with an increased risk of stroke (16 studies; RR, 1.49; 95% CI, 1.23–1.80; *P* < 0.001; *I*^2^ = 96%) (Figure 3). Subgroup analysis by study design showed similar results in both prospective (10 studies; RR, 1.53; 95% CI, 1.23–1.90; *P* < 0.001; *I*^2^ = 61%) and retrospective cohorts (6 studies; RR, 1.40; 95% CI, 1.06–1.85; *P* = 0.02; *I*^2^ = 98%), with heterogeneity being lower in the prospective group (Supplementary Table 4 and Supplementary Figure 4). When stratified by stroke subtype (Supplementary Figure 5), the pooled association was significant for ischemic stroke (7 studies; RR, 1.44; 95% CI, 1.22–1.70; *P* < 0.001; *I*^2^ = 79%) and for mixed types (8 studies; RR, 1.40; 95% CI, 1.04–1.88; *P* = 0.03; *I*^2^ = 95%).

**Figure 4:**
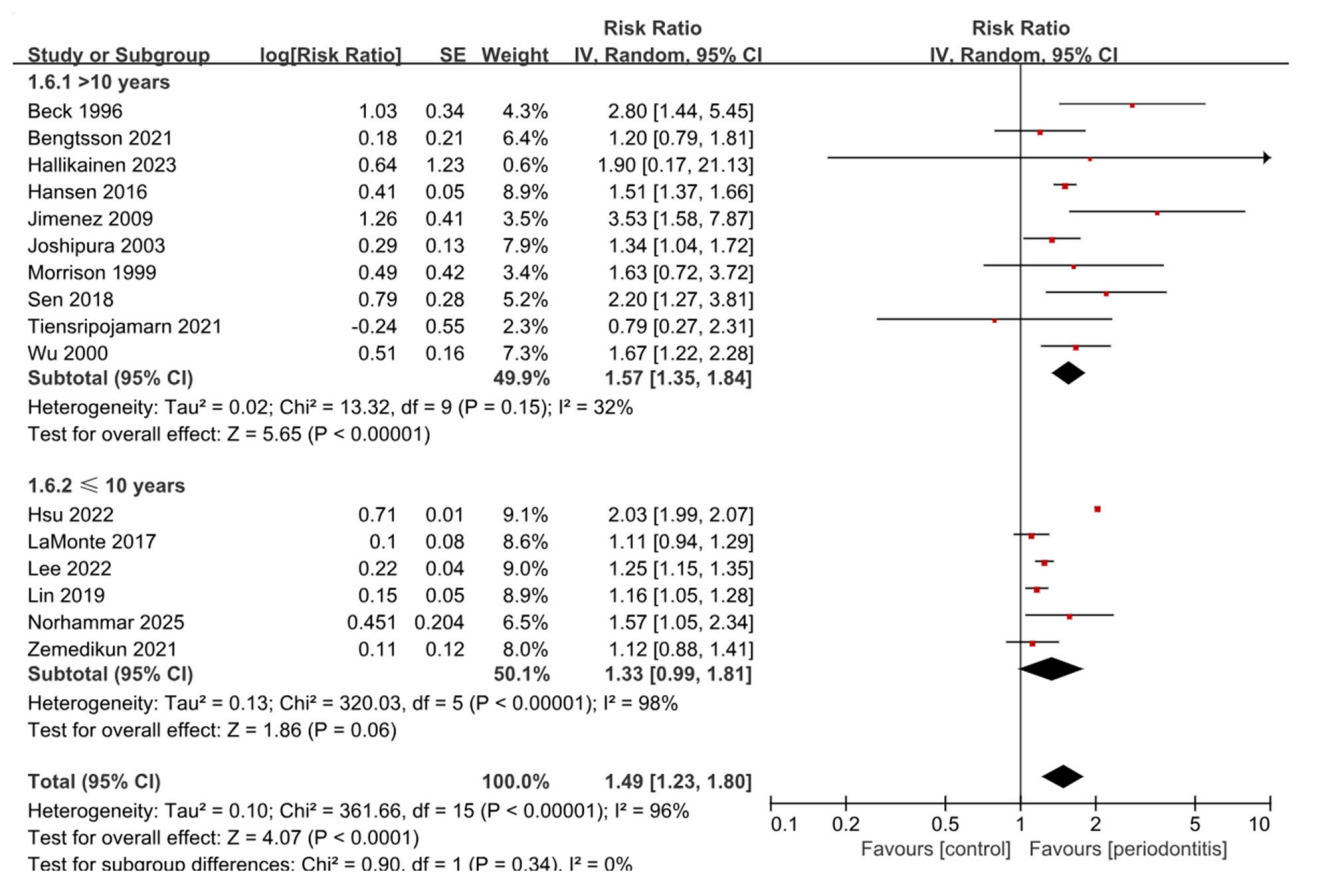
Subgroup forest plot by follow-up duration in cohort studies.

Notably, subgroup analysis by follow-up duration (Figure 4) highlighted a more stable association in studies with longer observation. Among 10 studies with follow-up longer than 10 years, periodontitis was associated with a 57% increased risk of stroke (RR, 1.57; 95% CI, 1.35–1.84; *P* < 0.001), with only moderate heterogeneity (I^2^ = 32%). By contrast, studies with ≤ 10 years of follow-up showed a weaker and nonsignificant association (6 studies; RR, 1.33; 95% CI, 0.99–1.81; *P* = 0.06; *I*^2^ = 98%). Subgroup analysis by participant sex is presented in Supplementary Figure 6.

Sensitivity analysis limiting to studies with low risk of bias did show the above results were stable. An additional sensitivity analysis excluding studies that used ICD codes or questionnaires to define periodontitis (Supplementary Figure 7) resulted in a slightly increased and less heterogeneous association between periodontitis and stroke in cohort studies (9 studies; RR, 1.53; 95% CI, 1.33–1.77; *P* < 0.001; *I*^2^ = 31%). This suggests that studies with more standardized diagnostic criteria for periodontitis provide more robust evidence. Other sensitivity analysis are presented in Supplementary Figures 8–10.

### Publication bias

Assessment of publication bias was performed only for cohort studies, as more than 10 studies were included in the meta-analysis. Visual inspection of the funnel plot did not suggest marked asymmetry (Supplementary Figure 11). Egger's regression test indicated a potential bias (*p* = 0.04). The Trim and Fill method (Supplementary Figure 12) imputed two missing smal studies. After adjustment, the pooled effect size was RR 1.40 (95% CI, 1.16–1.68), showing only a slight change from the original estimate, indicating minimal impact of publication bias.

## Discussion

In this systematic review and meta-analysis of 22 studies, periodontitis was associated with higher stroke risk. The pooled effects were OR 2.22 (95% CI, 1.48–3.34; *I*^2^ = 33%) in case–control studies and RR 1.49 (1.23–1.80; *I*^2^ = 96%) in cohorts. Studies with >10-year follow-up showed a more stable estimate (RR 1.57; 1.35–1.84; *I*^2^ = 32%). Our review extends earlier work by including more recent studies and prespecifying subgroup analyses to explore sources of heterogeneity.

Our findings are broadly consistent with prior syntheses but refine the magnitude and certainty. The ischemic-stroke meta-analysis by Leira et al. (37) reported larger pooled effects (cohort RR 2.52; case–control RR 3.04), likely reflecting fewer studies and earlier exposure/outcome definitions than in our datasets. Fagundes et al. (10) similarly concluded that periodontitis increases stroke risk (overall RR 1.88; 95% CI, 1.55–2.29), especially for ischemic events (case–control RR 2.72; 95% CI, 2.00–3.71). By contrast, a 2023 cardiovascular meta-analysis that included stroke reported a smaller association (RR 1.26; 95% CI, 1.15–1.37), aligning with our more conservative cohort estimate and suggesting that broader, later cohorts tend to attenuate effects (38). A recent study by Asmat-Abanto et al. (39) found that periodontitis was a risk factor for ischemic stroke (overall OR 2.59; 95% CI, 1.90–3.53). The authors included only 16 primary studies and noted significant heterogeneity even after subgrouping. Evidence regarding hemorrhagic stroke remains sparse and inconclusive, reinforcing separation by stroke type in evidence appraisal (40). Our study adds to this literature by including a larger number of studies and multiple prespecified subgroup analyses.

Biological pathways plausibly link periodontitis to stroke. Chronic oral dysbiosis sustains systemic inflammation (CRP, IL-6, TNF-α), impairs endothelial function, and promotes thrombosis; *P. gingivalis* gingipains can activate platelets and coagulation, providing a bridge to atherosclerotic events (41). Contemporary work on the oral–gut–brain axis further outlines how periodontal pathogens or their products (e.g., LPS, outer-membrane vesicles) translocate via blood or vagal routes, seed gut dysbiosis, and skew immune homeostasis, thereby amplifying cerebrovascular vulnerability (6). Experimental and translational studies indicate that *P. gingivalis* and *F. nucleatum* can promote atherosclerotic plaque and alter blood–brain barrier integrity, supporting a chain from periodontal inflammation to ischemic injury (42, 43). Genetic evidence adds specificity: Mendelian randomization suggests a potential causal effect of chronic periodontitis on cardioembolic stroke, consistent with a pro-thrombotic/inflammatory pathway (44). These lines of evidence are consistent with our stronger association in ischemic outcomes and in studies with longer follow-up.

Our subgroup analyses show a clearer pattern by study design: prospective cohorts yielded slightly higher and more stable estimates than retrospective studies (prospective RR = 1.53, 95% CI 1.23–1.90; *I*^2^ = 61% vs. retrospective RR = 1.40, 1.06–1.85; *I*^2^ = 98%). Longer observation strengthened this pattern (>10-year follow-up RR = 1.57, 1.35–1.84; *I*^2^ = 32%), supporting temporality and echoing earlier calls for better prospective evidence. By contrast, stroke subtype effects were broadly comparable in magnitude in our data (ischemic RR 1.44, 95%CI 1.22–1.70; mixed-type RR 1.40, 95%CI 1.04–1.88), whereas hemorrhagic stroke evidence remained sparse and imprecise, consistent with a recent focused review (40). These design-related differences likely reflect measurement and timing: retrospective cohorts often rely on single ICD codes or self-report and cannot track periodontal change, whereas prospective designs define exposure before the event and better capture latency (45). The stronger and more stable estimate with >10 years of follow-up argues against short-term reverse causation and is compatible with a slow process in which chronic inflammation and pro-thrombotic changes accumulate (46). Although the magnitudes for ischemic and other stroke were similar in our estimates, recent work suggests that not all ischemic subtypes behave the same: the association appears clearer for lacunar infarction with very low heterogeneity, which supports a small-vessel pathway (39). The higher estimate in male-only analyses should be interpreted with caution; residual confounding by smoking or socioeconomic status may remain even after adjustment.

Much of the between-study variation likely arises from how periodontitis and stroke were measured. Some cohorts used a single ICD code or self-report, while others used full-mouth clinical charts or radiographs. Newer European Federation of Periodontology/American Academy of Periodontology (EFP/AAP) 2018 criteria also differ from earlier case definitions and may change case finding. These differences can cause non-differential misclassification and pull effects toward the null (47, 48). Outcome ascertainment (incident vs. prevalent stroke; imaging vs. codes) and uneven adjustment for smoking, diabetes, BMI, socioeconomic status, and oral-hygiene behaviors may further contribute to heterogeneity. Publication bias appeared limited in our data, consistent with a null intercept on Egger's test (49).

For clinicians, periodontitis should be considered a modifiable associated factor in vascular risk management, especially in patients at high ischemic risk. Interventional studies show that periodontal therapy can improve endothelial function; a recent meta-analysis reported consistent gains in flow-mediated dilation after treatment (50). New trial data suggest that intensive periodontal therapy may slow carotid intima–media thickening, supporting a vascular mechanism, although longer multicenter trials are needed (51). Observational data from a nationwide Korean database further indicate that regular dental scaling is linked with lower stroke incidence among patients with moderate-to-severe periodontal disease (52). These findings align with our stronger association in long follow-up cohorts, but they should not be over-interpreted as proof of causality.

Future research should standardize and repeat periodontal assessments using the 2018 AAP/EFP framework, use incident stroke endpoints confirmed by imaging or reliable registries, and predefine stroke subtypes—including small-vessel disease—given the clearer lacunar signal in recent reviews. It should also test whether periodontal treatment reduces vascular events through randomized or quasi-experimental studies, together with Mendelian randomization and mediation analyses to clarify inflammatory and endothelial pathways.

In addition, future research should also consider exploring the relationship between gum disease and stroke outcomes. Specifically, it would be valuable to investigate whether periodontitis influences the prognosis of stroke patients after interventions such as thrombectomy, tissue plasminogen activator treatment, or craniotomy. Examining the potential impact of periodontitis on recovery and long-term health outcomes in stroke patients could provide important insights into its clinical relevance.

Our review has several limitations. First, heterogeneity was high in cohort syntheses (*I*^2^ up to 96%), reflecting wide variation including periodontitis definitions, teeth/sites examined, follow-up duration, and stroke ascertainment. Excluding studies with less precise diagnostic criteria (e.g., ICD codes, questionnaires) significantly reduced heterogeneity, highlighting that diagnostic variability is a major source of heterogeneity. Second, exposure misclassification is likely because most cohorts measured periodontal status once and did not track progression; non-differential error would bias effects toward the null, while differential error cannot be excluded. Third, residual confounding is unavoidable: adjustment sets differed across studies, and smoking, diabetes, BMI, socioeconomic status, and oral-hygiene behaviors were not uniformly controlled. Fourth, some effect measures required conversion (e.g., OR to RR for rare events); although results were robust in sensitivity analyses, model choice and rare-event handling may affect magnitude. Fifth, certain subgroups were underpowered (hemorrhagic stroke, female-only cohorts). Sixth, we analyzed study-level data rather than individual participant data, so consistent covariate adjustment and mediation testing were not possible. Finally, the review was not prospectively registered, which may introduce selection or reporting bias despite our predefined protocol and team adjudication. However, our study has strengths: this study comprised an ever large number of studies comparing with previous systematic review and meta-analysis. The pooled sample was large and geographically diverse.

## Conclusion

Periodontitis is associated with a higher risk of stroke in observational studies, with more stable estimates in long-term prospective cohorts. The association appears most evident for ischemic outcomes and is biologically plausible through inflammatory, endothelial, and pro-thrombotic pathways. Given high heterogeneity and potential residual confounding, these findings should be interpreted with caution. Standardized exposure definitions, subtype-specific endpoints, and well-designed interventional and causal studies are needed to determine whether improving periodontal health can reduce stroke risk.

## Data Availability

The raw data supporting the conclusions of this article will be made available by the authors, without undue reservation.
