# Supplementary material for: Periodontitis and risk of stroke: a systematic review and meta-analysis of observational studies
Source: Front Neurol. 2025 Nov 3;16:1700946. doi: 10.3389/fneur.2025.1700946 (PMC12620239; doi:10.3389/fneur.2025.1700946)
Supplement: Supplementary file 1 [file Table_1.docx]

**eMethods Search Strategy**
**Medline** through Pubmed:
#1. ("Stroke"[Mesh] OR "Brain Ischemia"[Mesh] OR "Stroke, Lacunar"[Mesh] OR Stroke[Title/Abstract] OR Strokes[Title/Abstract] OR "Cerebrovascular Accident*"[Title/Abstract] OR "Cerebral Stroke*"[Title/Abstract] OR "Ischemic Stroke*"[Title/Abstract] OR "Brain Ischemia*"[Title/Abstract] OR "Cerebral Ischemia*"[Title/Abstract] OR "Lacunar Stroke*"[Title/Abstract] OR "Lacunar Infarct*"[Title/Abstract] OR "Cerebrovascular Stroke*"[Title/Abstract]) 466,144

#2. ("Periodontitis"[Mesh] OR "Periodontal Diseases"[Mesh] OR "Alveolar Bone Loss"[Mesh] OR periodontitis[Title/Abstract] OR "chronic periodontitis"[Title/Abstract] OR "aggressive periodontitis"[Title/Abstract] OR "periapical periodontitis"[Title/Abstract] OR "periodontal disease*"[Title/Abstract] OR "periodontal bone loss"[Title/Abstract] OR "alveolar bone loss"[Title/Abstract] OR parodontosis[Title/Abstract] OR parodontoses[Title/Abstract] OR "pyorrhea alveolaris"[Title/Abstract]) 123,402

#3. #1 and #2 612

**Embase**
#1. 'stroke'/exp OR 'stroke' OR 'cerebrovascular accident'/exp OR 'cerebrovascular accident' OR 'brain ischemia'/exp OR 'brain ischemia' OR 'ischemic stroke'/exp OR 'ischemic stroke' OR 'lacunar infarction'/exp OR 'lacunar infarction' 890,631

#2. stroke*:ti,ab,kw OR 'cerebrovascular accident*':ti,ab,kw OR 'cerebral infarct*':ti,ab,kw OR 'ischemic stroke*':ti,ab,kw OR 'brain ischem*':ti,ab,kw OR 'cerebral ischem*':ti,ab,kw OR 'lacunar stroke*':ti,ab,kw OR 'lacunar infarct*':ti,ab,kw OR ((cerebrovascular NEAR/3 accident*):ti,ab,kw) OR ((brain NEAR/3 infarct*):ti,ab,kw) OR ((cerebral NEAR/3 ischem*):ti,ab,kw) 675,979

#3. #1 OR #2 922,820

#4. 'periodontitis'/exp OR 'periodontal disease'/exp OR 'alveolar bone loss'/exp 147,736

#5. periodontitis:ti,ab,kw OR 'chronic periodontitis':ti,ab,kw OR 'aggressive periodontitis':ti,ab,kw OR 'periapical periodontitis':ti,ab,kw OR 'periodontal disease*':ti,ab,kw OR 'periodontal bone loss':ti,ab,kw OR 'alveolar bone loss':ti,ab,kw OR parodontosis:ti,ab,kw OR parodontoses:ti,ab,kw OR 'pyorrhea alveolaris':ti,ab,kw 68,818

#6. #4 OR #5 158,455

#7. #3 AND #6 1,921

**Cochrane Database of Systematic Reviews**

#1 (stroke OR strokes OR "cerebrovascular accident*" OR "cerebrovascular apoplexy" OR "cerebral infarct*" OR "cerebral stroke*" OR "cerebrovascular stroke*" OR "brain ischem*" OR "cerebral ischem*" OR "ischemic stroke*" OR "lacunar stroke*" OR "lacunar infarct*" OR "lacunar infarction*" OR "acute stroke*" OR "acute cerebrovascular accident*"):ti,ab,kw 81215

#2 (periodontitis OR "chronic periodontitis" OR "aggressive periodontitis" OR "periapical periodontitis" OR "periodontal disease*" OR "periodontal diseases" OR "periodontal bone loss" OR "alveolar bone loss" OR parodontosis OR parodontoses OR "pyorrhea alveolaris"):ti,ab,kw 12261

#3 #1 AND #2 61

**Table 1.** Quality assessment of included cohort studies via Newcastle-Ottawa Scale.

| **Study** | **Is the case  definition  adequate?** | **Representativeness of the  cases** | **Selection of  controls** | **Definition of  controls** | **Comparability  of cases and controls based on the  design or analysis** | **Ascertainment  of exposure** | **The same  method of ascertainment  for cases and  controls** | **Non-response rate** |
| --- | --- | --- | --- | --- | --- | --- | --- | --- |
| Beck 1996 | * | * | * | * | ** | * | * | - |
| Bengtsson 2021 | * | * | * | * | ** | * | * | * |
| Hallikainen 2023 | * | * | * | * | * | * | * | * |
| Hansen 2016 | * | * | * | * | ** | * | * | * |
| Hsu 2022 | * | * | * | * | ** | * | * | * |
| Jimenez 2009 | * | * | * | * | ** | * | * | - |
| Joshipura 2003 | * | * | - | * | ** | * | * | * |
| LaMonte 2017 | * | * | - | * | ** | * | * | * |
| Lee 2022 | * | * | * | * | ** | * | * | * |
| Lin 2019 | * | * | * | * | ** | * | * | * |
| Morrison 1999 | * | * | * | * | ** | * | * | - |
| Norhammar 2025 | * | * | * | * | ** | * | * | * |
| Sen 2018 | * | * | * | * | ** | * | * | * |
| Tiensripojamarn 2021 | * | * | * | * | ** | * | * | - |
| Wu 2000 | * | * | * | * | ** | * | * | * |
| Zemedikun 2021 | * | * | * | * | ** | * | * | * |

**Table 2.** Quality assessment of included case control studies via Newcastle-Ottawa Scale.

| **Study** | **Is the case  definition  adequate?** | **Representative-ness of the  cases** | **Selection of  controls** | **Definition of  controls** | **Comparability  of cases and controls based on the  design or analysis** | **Ascertainment  of exposure** | **The same  method of ascertainment  for cases and  controls** | **Non-response rate** |
| --- | --- | --- | --- | --- | --- | --- | --- | --- |
| Abolfazli 2011 | * | * | * | * | ** | - | * | * |
| Diouf 2015 | * | * | * | - | ** | * | * | * |
| Dörfer 2004 | * | * | * | * | ** | * | * | - |
| Grau 2004 | * | * | * | * | ** | * | * | * |
| Hashemipour 2013 | * | * | * | * | ** | * | * | - |
| Pradeep 2010 | * | * | - | * | ** | * | * | * |

**Table 3.** Subgroup analysis restricted to case-control studies**.**

|  | **Studies** | **Effect size** | **95%CI** | ***P* value** | **I2** |
| --- | --- | --- | --- | --- | --- |
| Sex |  |  |  |  |  |
| Female | 0 | - | - | - | - |
| Male | 0 | - | - | - | - |
| Mixed | 6 | 2.22 | 1.48 to 3.34 | <0.01 | 33% |
| Study design |  |  |  |  |  |
| Prospective | 0 | - | - | - | - |
| Retrospective | 6 | 2.22 | 1.48 to 3.34 | <0.01 | 33% |
| Mean follow-up duration |  |  |  |  |  |
| > 10 years | - | - | - | - | - |
| ≤10 years | - | - | - | - | - |
| Stroke subtype |  |  |  |  |  |
| Ischemic | 5 | 2.66 | 1.65 to 4.28 | <0.01 | 12% |
| Hemorrhagic | 0 | - | - | - | - |
| Mixed | 1 | 1.58 | 1.11 to 2.25 | 0.01 | - |

**Table 4.** Subgroup analysis restricted to cohort studies**.**

|  | **Studies** | **Effect size** | **95%CI** | ***P* value** | **I2** |
| --- | --- | --- | --- | --- | --- |
| Sex |  |  |  |  |  |
| Female | 1 | 1.11 | 0.94 to 1.29 | 0.02 | - |
| Male | 3 | 2.18 | 1.13 to 4.24 | 0.21 | 76% |
| Mixed | 12 | 1.44 | 1.16 to 1.79 | <0.01 | 96% |
| Study design |  |  |  |  |  |
| Prospective | 10 | 1.53 | 1.23 to 1.90 | <0.01 | 61% |
| Retrospective | 6 | 1.40 | 1.06 to 1.85 | 0.02 | 98% |
| Mean follow-up duration |  |  |  |  |  |
| > 10 years | 10 | 1.57 | 1.35 to 1.84 | <0.01 | 32% |
| ≤10 years | 6 | 1.33 | 0.99 to 1.81 | 0.06 | 98% |
| Stroke sub-type |  |  |  |  |  |
| Ischemic | 7 | 1.44 | 1.22 to 1.70 | <0.01 | 79% |
| Hemorrhagic | 1 | 1.90 | 0.17 to 21.13 | 0.60 | - |
| Mixed | 8 | 1.40 | 1.04 to 1.88 | 0.03 | 95% |


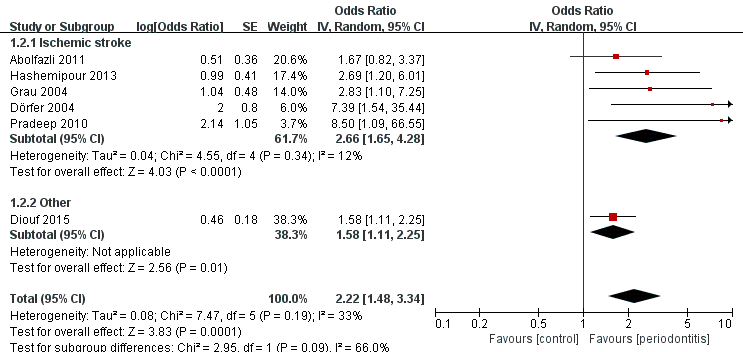


**Figure 1**. Subgroup analysis by stroke sub-type in case-control studies.


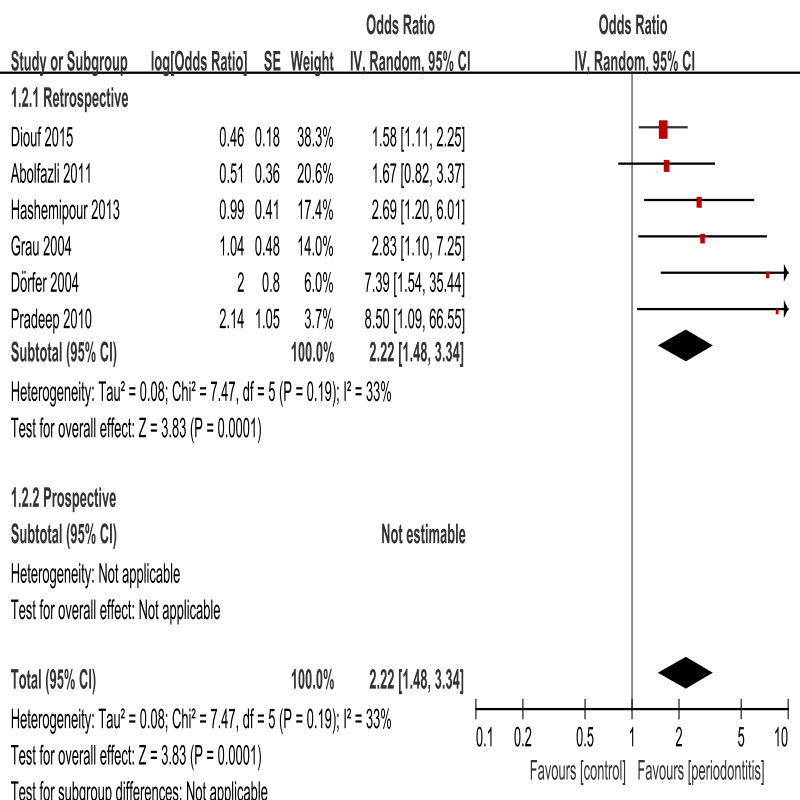


**Figure 2**. Subgroup analysis by study design in case-control studies.


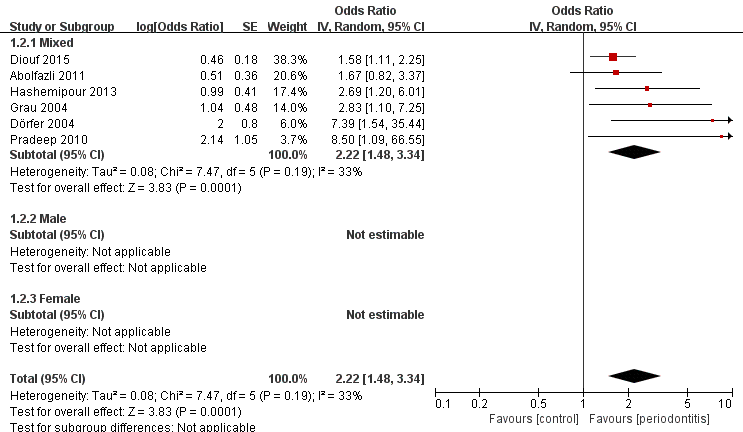


**Figure 3.** Subgroup analysis by participant sex in case-control studies.


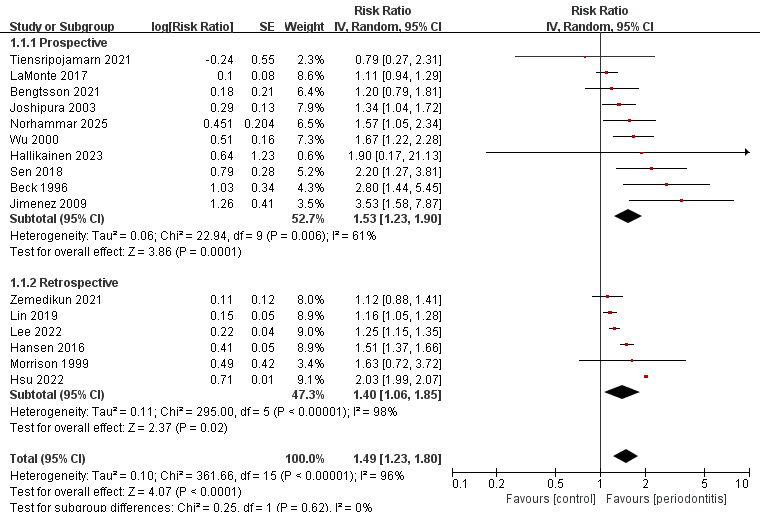


**Figure 4.** Subgroup analysis by study design in cohort studies.


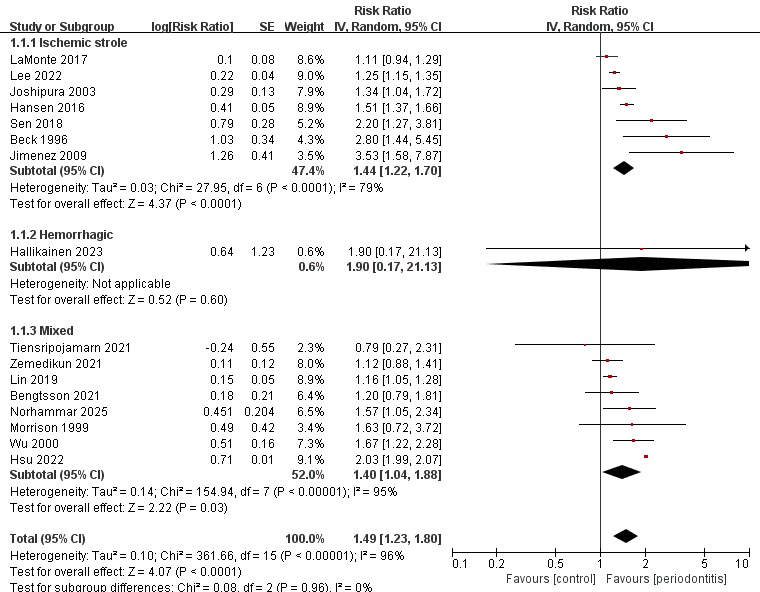


**Figure 5.** Subgroup analysis by stroke sub-type in cohort studies.


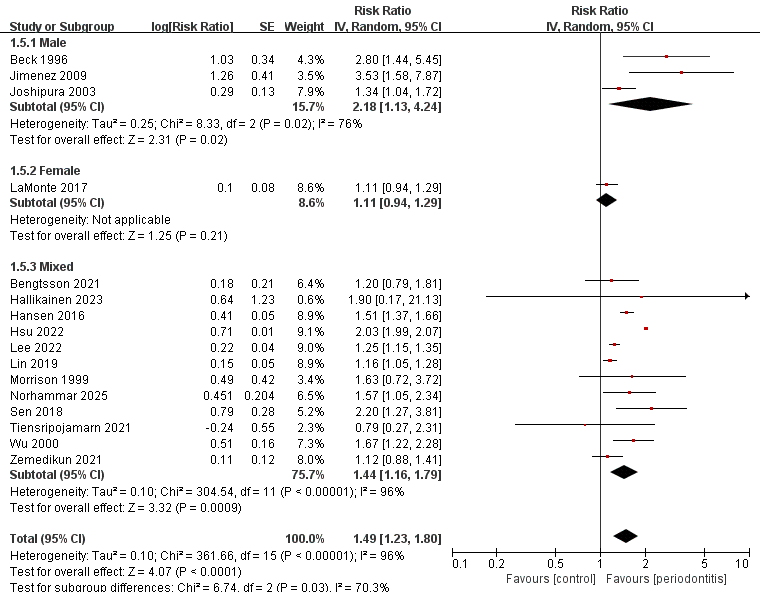


**Figure 6**. Subgroup analysis by participant sex in cohort studies.

**
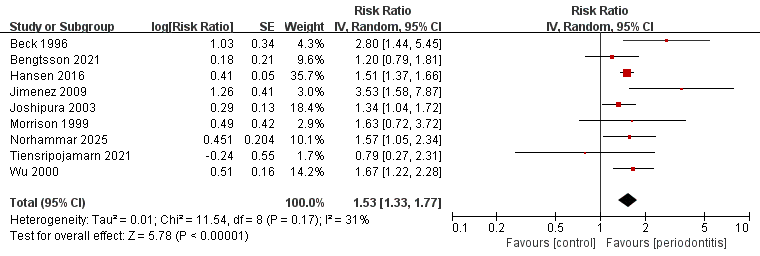
**

**Figure 7.** Sensitivity analysis by restricting to studies with periodontitis diagnosis through clinical examination or radiographic assessment in cohort studies.

**
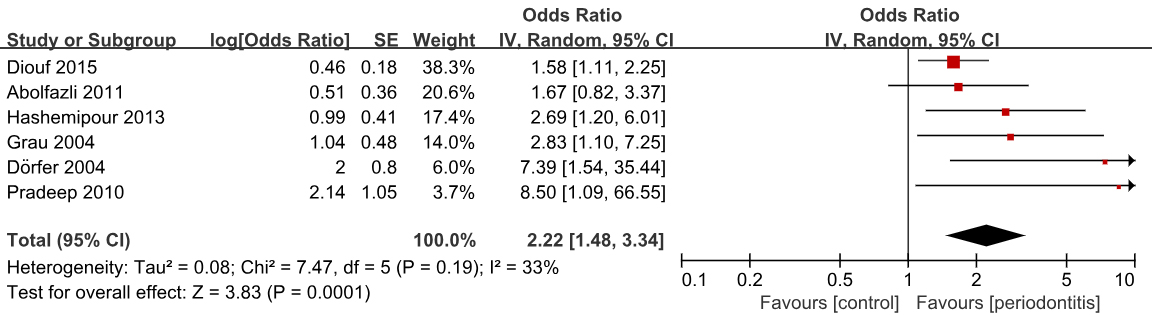
**

**Figure 8.** Sensitivity analysis by restricting to studies with periodontitis diagnosis through clinical examination or radiographic assessment in case-control studies.


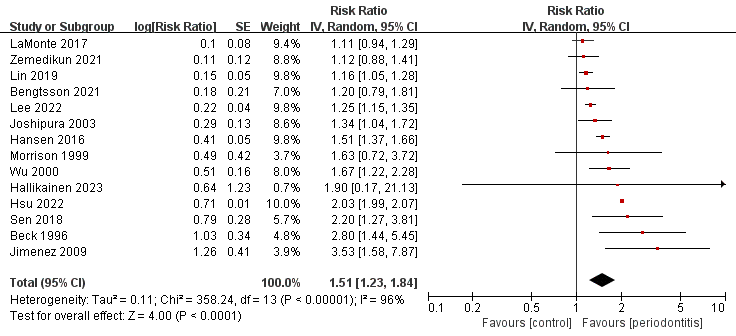


**Figure 9.** Sensitivity analysis by restricting to studies with multivariable-adjusted estimates in cohort studies.


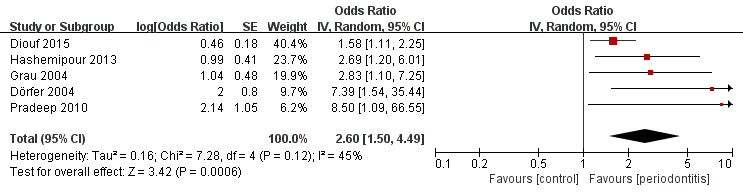


**Figure 10.** Sensitivity analysis by restricting to studies with multivariable-adjusted estimates in case-control studies.

**
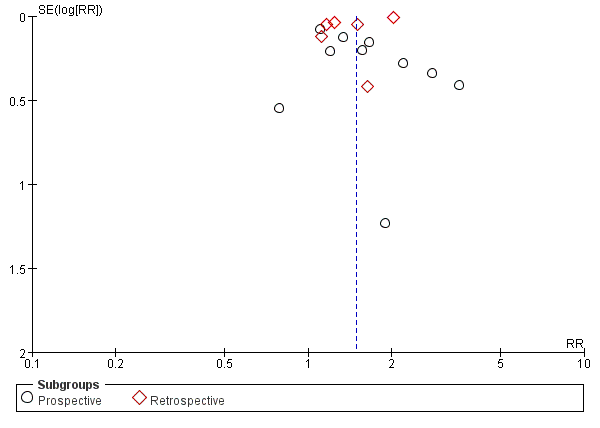
**

**Figure 11.** Funnel plot of cohort studies.


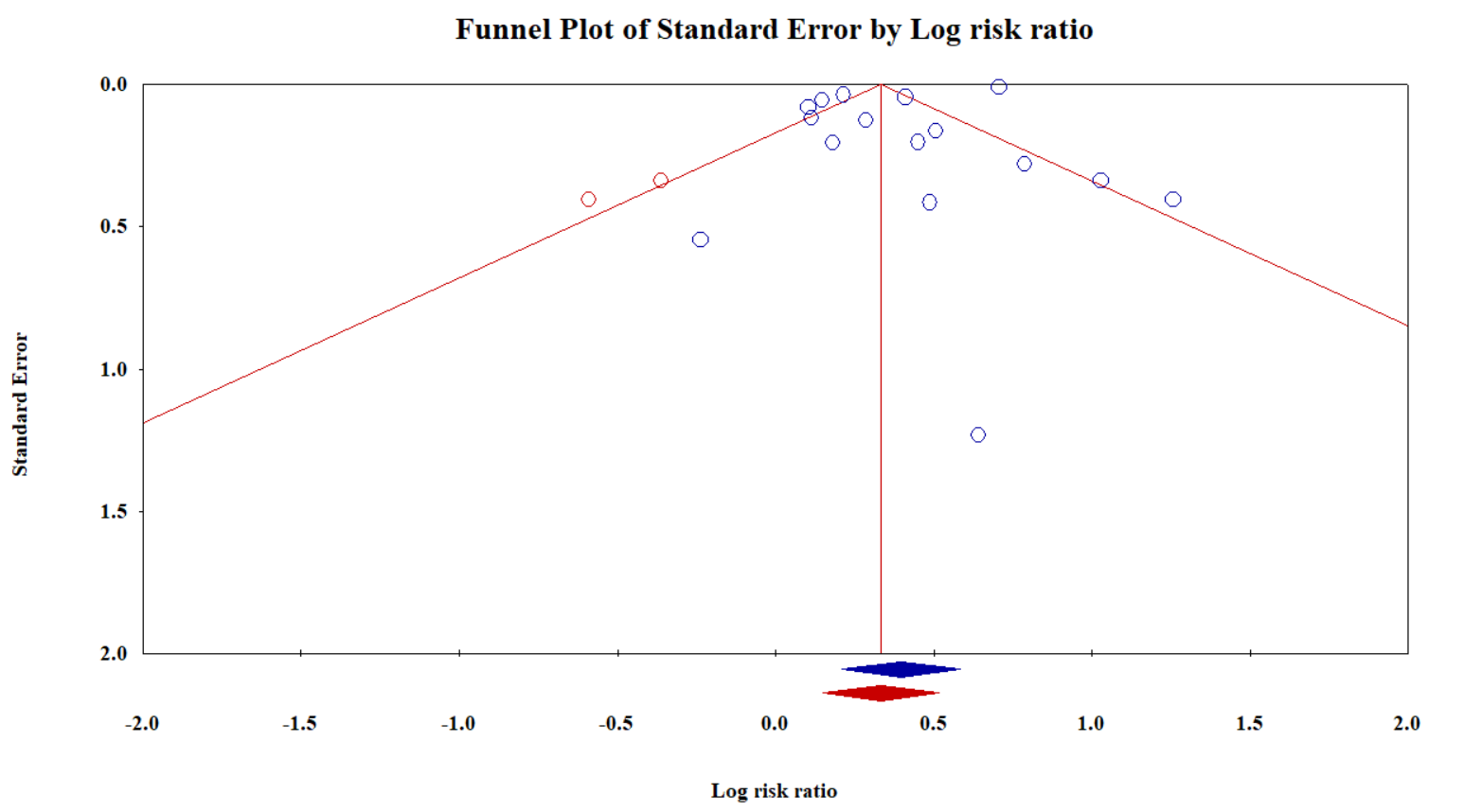


**Figure 12.** Funnel plot of observed and imputed cohort studies.
